# Genomic Variants Associated with Resistance to High Fat Diet Induced Obesity in a Primate Model

**DOI:** 10.1038/srep36123

**Published:** 2016-11-04

**Authors:** R. Alan Harris, Callison E. Alcott, Elinor L. Sullivan, Diana Takahashi, Carrie E. McCurdy, Sarah Comstock, Karalee Baquero, Peter Blundell, Antonio E. Frias, Maike Kahr, Melissa Suter, Stephanie Wesolowski, Jacob E. Friedman, Kevin L. Grove, Kjersti M. Aagaard

**Affiliations:** 1 Department of Obstetrics & Gynecology, Division of Maternal-Fetal Medicine at Baylor College of Medicine and Texas Children’s Hospital, Houston, TX, USA; 2 Department of Molecular and Human Genetics at Baylor College of Medicine, Houston, TX, USA; 3 Developmental Biology Interdisciplinary Program at Baylor College of Medicine, Houston, TX, USA; 4 Oregon National Primate Research Center, Oregon Health & Science University (OHSU), Beaverton, OR, USA; 5 Department of Biology, University of Portland, USA; 6 Department of Human Physiology, University of Oregon, Eugene, OR, USA; 7 Department of Biology, Corban University, Salem, OR, USA; 8 Department of Obstetrics & Gynecology, Division of Maternal-Fetal Medicine, OHSU, Portland, OR, USA; 9 Departments of Pediatrics, University of Colorado Anschutz Medical Campus, Aurora, CO, USA; 10 Department of Molecular and Cell Biology at Baylor College of Medicine, Houston, TX, USA

## Abstract

Maternal obesity contributes to an increased risk of lifelong morbidity and mortality for both the mother and her offspring. In order to better understand the molecular mechanisms underlying these risks, we previously established and extensively characterized a primate model in *Macaca fuscata* (Japanese macaque). In prior studies we have demonstrated that a high fat, caloric dense maternal diet structures the offspring’s epigenome, metabolome, and intestinal microbiome. During the course of this work we have consistently observed that a 36% fat diet leads to obesity in the majority, but not all, of exposed dams. In the current study, we sought to identify the genomic loci rendering resistance to obesity despite chronic consumption of a high fat diet in macaque dams. Through extensive phenotyping together with exon capture array and targeted resequencing, we identified three novel single nucleotide polymorphisms (SNPs), two in apolipoprotein B (APOB) and one in phospholipase A2 (PLA2G4A) that significantly associated with persistent weight stability and insulin sensitivity in lean macaques. By application of explicit orthogonal modeling (NOIA), we estimated the polygenic and interactive nature of these loci against multiple metabolic traits and their measures (*i.e*., serum LDL levels) which collectively render an obesity resistant phenotype in our adult female dams.

Obesity results from a complex set of interactions between genetics and the environment, and links behavior, metabolism and early in life exposure modeling. In the last several decades, a global epidemic of increasing rates of obesity in humans has been well described but relatively poorly understood at a genomic level^1^. Obesity is known to be heritable, with the most recent in a wave of meta-analyses of body mass index (BMI) genome-wide association studies (GWAS) estimating that 97 loci account for approximately 2.7% of BMI variation, and common variants account for up to 21% of BMI variation^2^. However, these loci only nominally predict obesity (defined as BMI ≥30 kg per m^2^), as measured by an improved area under the receiver-operating characteristic curve from 0.576 to 0.601 in a model including age, sex, and four genotype-based principal components^2^. Similarly, GWAS meta-analysis focused on the waist-to-hip ratio (WHR) as a measure of body fat distribution identified 20 loci with female sexual dimorphism which were associated with WHR adjusted for BMI^3^. Notably, these loci generally mapped to genes expressed in mesenchymal derived tissues that are linked to fat distribution and central obesity. These studies are consistent with prior equally robust GWAS analyses, alongside family, twin and adoption studies, and collectively suggest that a wide range (e.g., 40–70%) of inter-individual variability in BMI may be attributed to genetic factors, with *FTO* and near-*MC4R* loci seeming to affect obesity-susceptibility across a number of ancestries^4^,^5^,^6^,^7^,^8^,^9^,^10^,^11^,^12^,^13^,^14^. However, these studies also suggest that the majority of occurrences of obesity- or adiposity-deposition related traits cannot be attributed solely to inheritance of risk-laden genomic variants given their low minor allele frequency (<5%) and relatively modest effect size estimates (per allele OR ranging from 1.2–1.6) in the face of the current high prevalence of obesity^2^,^12^,^15^.

Given such relative rarity (or, alternately, low penetrance) of obese susceptibility variants in the general population, but persistent evidence for heritability^12^, it is possible that an equal, if not greater, magnitude of inter-individual BMI variance may arise from obese resistant loci. While it may be argued that this is merely “two sides of the same coin”, identification of loci which render relative resistance to obesity could explain the current prevalence observed in nearly all human population cohorts. In other words, given that a near majority of the human population is obese, identification of obesity resistant loci is likely to be of higher yield. However, identification of potential obesity resistance loci necessitates use of cohorts where chronic caloric excess with strong phenotypic profiling is well detailed. Because such studies cannot be practically carried out in human populations, robust identification of obese resistance loci necessitate relevant animal models. Indeed, initial evidence of loci mediating obesity resistance (but not sensitivity) exists in mice^16^,^17^, and have revealed associations with pathways that govern appetite, energy expenditure and food selection. The rare occurrence of such resistant loci over time would explain the current prevalence of obesity on a global scale, since their anticipated minor allele frequency would effectively render the large majority of the population as vulnerable to obesity. Moreover, because of the inherent heterogeneity of potentiating factors in human populations (*i.e.,* diet, exercise, early life exposures, reproductive life-stage, sex, and co-morbid metabolic conditions), identifying clearly defined obese susceptible and resistant genomic variants in humans is exceedingly challenging since it necessitates enormous subject cohorts in ancestrally diverse populations with reliable linked exposure data for fine-mapping of loci.

An alternative to human meta-analyses of GWAS data are strong phenotype-genotype studies in relevant non-human primate models. To this end, we have spent nearly a decade establishing and characterizing the effect of obesity and chronic high fat dietary exposure on adult female dams and their offspring in *Macaca fuscata* (Japanese macaque)^18^,^19^,^20^,^21^. Specifically, we have demonstrated that a high fat diet (HFD) comprised of 36% calories from fat during gestation and lactation structures the offspring’s epigenome, metabolome, and intestinal microbiome^18^,^19^,^22^,^23^,^24^,^25^,^26^,^27^; these molecular and metabolic perturbations are accompanied by persistent changes in offspring behavior and impairments in critical neural circuitry despite weaning to a healthy diet^28^. Of interest, we have consistently observed that HFD feeding for 1–5 years prior to pregnancy leads to insulin resistance and obesity in the majority, but not all, exposed dams^18^,^23^,^26^,^27^,^29^,^30^,^31^. Approximately 1/3 of our female macaque dams will remain relatively lean and insulin sensitive despite up to 8 years of exposure to the HFD and multiple pregnancies. Similar studies in humans have defined this phenomena with respect to short term adaptations to overfeeding^32^,^33^. In this current study we utilized exon-hybrid capture array and sequencing to identify and ascribe genomic loci to traits of adult obesity resistance, relative weight stability, and insulin sensitivity following prolonged high fat diet exposure among female macaque dams in a well-developed primate model. Although there are potential limitations to exon-hybrid capture over GWAS analyses, given the absence of a full species genome assembly use of exon-hybrid capture enabled identification of several *de novo* SNPs of interest for subsequent validation in an inclusive larger cohort.

## Results

### Novel SNP Identification and Genotyping with an Exon-Hybrid Capture Array and Sequencing

To identify functional and potentially novel SNPs associated with sensitivity or resistance to the development of obesity with high fat diet consumption present in our well characterized Japanese macaque (*Macaca fuscata*) model^20^,^21^,^22^,^23^,^24^,^25^,^26^,^27^,^28^,^29^,^30^,^31^,^32^,^33^, we designed an exon capture array that enriches for targeted exonic segments with similarity to the array probes (Supplemental Fig. 1). Our choice of utilization of exon hybrid capture for identification of potentially novel SNPs was based on the absence of a *Macaca fuscata* gene assembly, and subsequent concern for misclassification of whole genome sequencing data which would be inherent to mapping onto a moderate (6–12X) resolution *Macaca mulatta* rhesus assembly. Genes were selected for inclusion on the array based on a role in obesity, hyperlipidemia, insulin resistance, or lipid metabolism (such as those for cholesterol and triglyceride metabolism). Since the *Macaca fuscata* genome has not been sequenced, we used the currently available primate genome assemblies, with an emphasis on the rhesus macaque (*M. mulatta*) genome which diverged from *M. fuscata* 0.31 to 0.88 million years ago^34^, to identify sequences for inclusion on the array. Our custom array contained 78,450 probes spanning 783 genes. Our adult colony is genetically stable, and has been closed for 5 decades. However, it is not inbred and the mean kinship among the 186 animals in the *M. fuscata* breeding group is a grand mean of 0.01802 (inner and outer quartile of 0.01342 and 0.02223, respectively).

For the naïve discovery experiment employing our exon array, we selected a total of 8 *M. fuscata* adult females exposed to the HFD for a minimum of 3 years. We used data from non-pregnant females collected during their third year on the HFD for trait classification, and classified-animals as resistant (n = 4) or sensitive (n = 4) based on mean % bodyweight gain (22.3 vs 66.5%; range −10.98 to 113.51%), leptin/body weight ratio (0.08 vs 0.79; range 0.07 to 1.12 ng/ml relative to kg body weight), and insulin AUC (2589 vs 10210 μU/ml; range 1307 to 16480 μU/ml). Genomic DNA was extracted, hybridized to the array, and sequenced using the 454 FLX Titanium platform. SNPs of potential further interest for validation were first called based on an odds ratio approximating infinity (*i.e.,* observed in all 4 obese resistant dams, but not in any of the sensitive animals, or in all 4 animals obese dams, but not in any of the resistant). These criteria were sequentially relaxed to allow for 3 of 4 and 2 of 4 in one cohort, but still absent in the comparison cohort (Supplemental Table S1).

**Figure 1:**
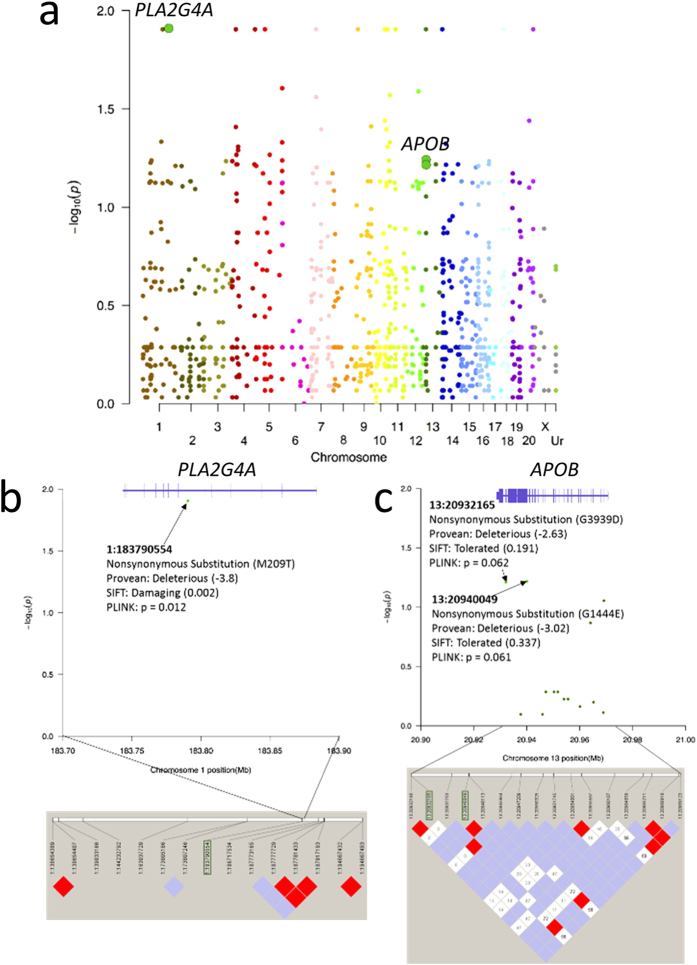
Manhattan plot (**a**) of exon sequencing identified SNPs compared between high fat diet sensitive and resistant Japanese macaques based on PLINK (initial ssahaSNP odds ratio approximating infinity, PLINK *p* of 0.012 to <0.063). −log p values are plotted on the y-axis, while chromosome location is plotted on the x-axis. Manhattan plots and linkage disequilibrium plots of SNPs in *PLA2G4A* (**b**) and *APOB* (**c**) identified as being putatively associated with lean versus obese response to chronic high fat diet in Japanese macaque dams.

To assure high quality SNP identification, we used three tools to call SNPs (Atlas-SNP2^35^, SNPTools^36^, and ssahaSNP^37^) and only selected the 1534 SNPs called by all three for further analysis (Supplemental Fig. 2 and Supplemental Tables S1 and S2). The SNPs were initially called using ssahaSNP^37^ (http://www.sanger.ac.uk/science/tools/ssahasnp) which only identifies homozygous alternative allele SNPs. Genes were identified for further analysis on Atlas-SNP2 and SNPTools if ssahaSNP made homozygous alternative allele calls in all the samples in one group, but none of the samples in the other group (odds ratio approximating infinity). Among the 1534 SNPs called by all three SNP calling tools, Variant Effect Predictor (VEP)^38^ based on rhesus gene models was used to identify functional consequences of the SNPs. SNPs were then lifted over to the human genome in order to perform SIFT^39^ and PROVEAN^40^ predictions of the effect of amino acid differences. PLINK^41^ was thereafter used to identify associations between the genotypes with HFD resistant or sensitive phenotypes (Fig. 1a). Nonsynonymous SNPs with SIFT or PROVEAN predicted effects on protein function, and PLINK adaptive permutation approaching genome wide significance (odds ratios approaching 2.0 to 2.5, which corresponded to an initial *p* value < 0.063 in our small discovery cohort) were chosen for further analysis. A SNP in phospholipase A2, group IVA (Cytosolic, Calcium-Dependent) (*PLA2G4A*) (p = 0.012) (Fig. 1b) and two SNPs in apolipoprotein B (*APOB*) (p = 0.061 and p = 0.062) (Fig. 1c) met these criteria.

The *PLA2G4A* and *APOB* SNPs were further interrogated by two means: functional annotation and validation in an expansive cohort. Functional annotation used a Combined Annotation Dependent Depletion (CADD)^42^ (Supplemental Table S3), which integrates multiple annotations to predict the likelihood of a SNP having a functional consequence. The *PLA2G4A* SNP had the highest PHRED scaled C-score (23.6). A PHRED score of ≥20 indicates the SNP is among the 1% of SNPs in the human genome most likely to have a functional effect. The PHRED scores for the *APOB* chr13:20932165 (9.018) and chr13:20940049 (15.07) approach or surpass a PHRED score ≥10 which indicates they are predicted to be among the 10% most likely SNPS to have a functional effect. Examination of individual constituents of the CADD scores showed that the *PLA2G4A* and *APOB* chr2:21235409 loci are highly conserved across primates, including humans.

In an effort to further identify significant associations with multiple additional traits that accompany or lend to the obesity resistant phenotype (*i.e.,* exploration of potential mechanisms rendering an association with obesity resistance in our cohort of strictly experimentally defined animals (*i.e.,* dams resistant to chronic high fat diet feeding for a minimum of 3 years), we took a dual approach. First, we completed expanded phenotyping among a total of 71 animals, inclusive of 43 adult female dams who had been placed on the HFD for at least 3 years and subjected to expansive phenotypic testing and measured analysis (the initial 8 dams, alongside 35 additional unrelated animals; *n* = 43 high fat diet challenged dams in total; 28 additional animals with partial phenotypic data were similarly included for a total of 71 animals). Second, the *PLA2G4A* and *APOB* SNPs were assayed by PCR based genotyping in an extended panel among all 43 adult female dams on the HFD for at least 3 years with extensive trait-correlated and trait-independent phenotypic data (Supplemental Table S4). In an effort to link genotype to the significantly expanded phenotype (as described further below), the extended SNP panel was designed to include the 8 individuals that were assayed by exon capture arrays along with their expanded trait data.

### Expanded Phenotyping and Trait Characterization

**Figure 2:**
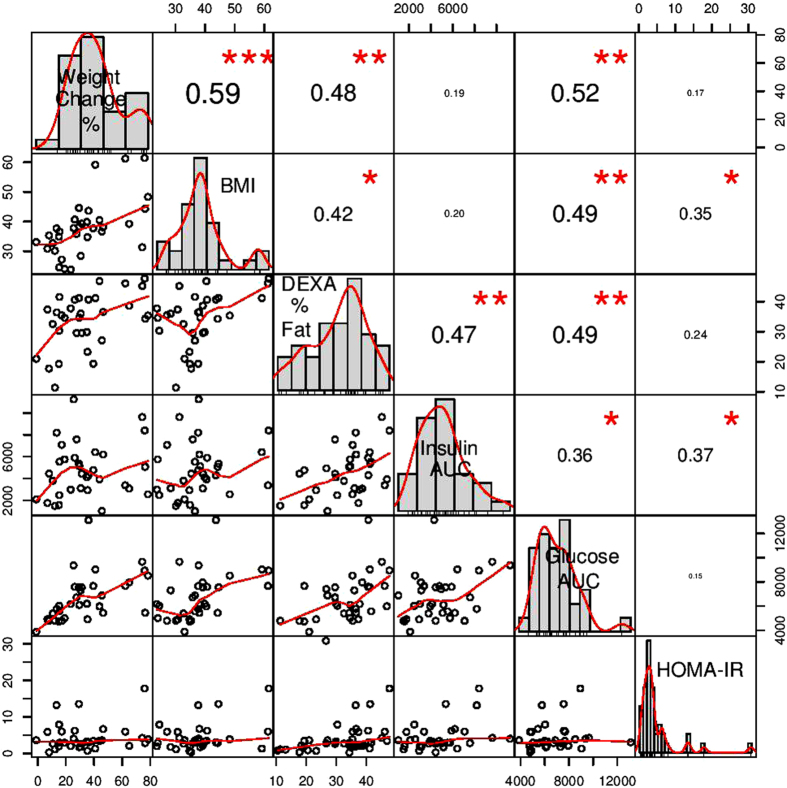
Scatterplot matrix of correlations among measured phenotypes for high fat diet exposed Japanese macaques. Only phenotypes that show significant genotype-phenotype associations based on NOIA are shown. The diagonal is labelled with the phenotypes and shows the distribution of the values for that phenotype. Scatterplots comparing two phenotypes together with red trend lines are shown below the diagonal. Pearson correlations for comparisons between two phenotypes are shown above the diagonal with significance denoted by (*p < 0.05), (**p < 0.01) and (***p < 0.001).

While human epidemiologic studies have used categorical definitions in units of kg/m^2^ for ascribing normal weight, overweight, and obesity classifications^43^, they are in fact continuous variables and both body weight and body composition traits are quantitative in nature. To account for the complexity and heterogeneity of relative body mass, we performed extensive phenotyping of 37 traits on the individuals in the extended trait panel, including weight, percent and distribution body fat as measured by Dual-energy X-ray Absorptiometry (DXA) scan, cholesterol, insulin, glucose, leptin, and glucagon measurements as well as aggregate functions of the measurements such as HOMA-IR (Supplemental Table S5). A scatterplot matrix of correlations between a selected subset of phenotypes that show significant genotype-phenotype associations are shown in Fig. 2. The significant correlations between many of the traits highlights the relatedness of the phenotypic measures that serves to define an individual signature of obesity and insulin sensitivity in response to chronic HFD feeding in adult females. By identifying traits which independently segregate and distinguishing them from those that are co-linear, we were able to differentiate genotypes with pleiotropic affects. The significant genotype-phenotype associations of these phenotypes with the SNPs we identified in *APOB* and *PLA2G4A* suggest that the genotypes have pleiotropic effects on the phenotypes.

At the time of initial allocation onto the HFD, adult animals subsequently classified as obese resistant and sensitive did not significantly differ by virtue of number of prior pregnancies (resistant: nulliparous *n* 5, multiparous *n* 12; sensitive: nulliparous *n* 4, multiparous *n* 20; *p* = 0.45), age starting diet (resistant, 5.2 years vs. sensitive, 6.0 years; *p* = 0.12) nor age at first pregnancy after starting diet (resistant 6.4 years vs. sensitive 7.3 years, *p* = 0.10), nor initial baseline metabolic phenotyping (Supplemental Table S5). Initial body weight did show a statistically but unlikely clinically significant difference (resistant 7.93 kg vs sensitive 9.06 kg; *p* = 0.02).

### Genotype-Phenotype Association

We first performed PLINK analysis to identify associations between the genotypes in our extended panel and the phenotypes. Case/control association analysis based on the initial sensitive/resistant phenotype classification did not show significant association with genotypes (Supplemental Table S6). Case/control analysis based on the revised sensitive/resistant classification showed a significant (*p* = 0.005) association between the classification and the *PLA2G4A* SNP. PLINK quantitative trait association analysis showed significant (*p* < 0.05) associations of both *APOB* SNPs with insulin and glucose AUC after year 3 on the HFD (Supplemental Table S7). The APOB.1 SNP showed a significant association with weight and weight change after 3 years on the HFD. In addition, the APOB.1 SNP showed a significant association with the initial BMI at the start of the diet and after 3 years on the HFD.

**Figure 3:**
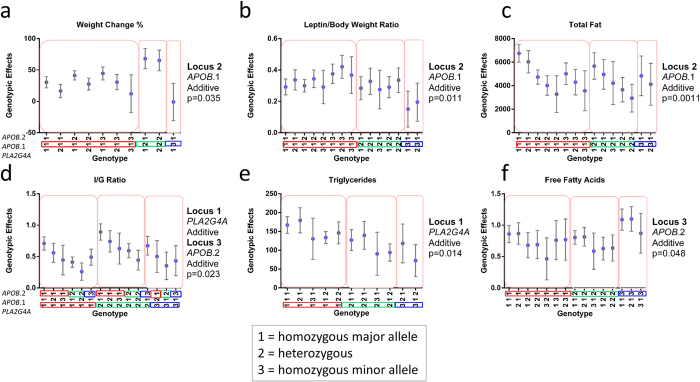
Selected genotype-phenotype NOIA three loci models based on genotypes from the extended panel of high fat diet exposed Japanese macaques. The Genotype (x-axis) shows the genotypes for each of the loci (ordered as *PLA2G4A*, *APOB*.1, and *APOB*.2) and the genotypes predicted to associate with the phenotype are outlined with colored boxes. The Genotypic Effects (y-axis) shows the NOIA predicted effect of the genotype on a phenotype. The *APOB.*1 locus demonstrated an additive effect on weight change percent (**a**), leptin/body weight ratio (**b**), and total fat based on DXA (**c**). (**d**) The *PLA2G4A* and *APOB*.2 loci show a combined additive effect on insulin/glucose ratio. (**e**) The *PLA2G4A* locus shows an additive effect on triglyceride levels. (**f**) The *APOB*.2 locus shows an additive effect on free fatty acid levels.

As a secondary analytic approach, we applied the Natural and Orthogonal InterAction (NOIA)^44^ model to the extended panel of SNPs suggested by PLINK analysis to refine genotype-phenotype associations. NOIA has the advantage of estimating interaction between genes (or epistasis), which is crucial in the determination of genomic variants in complex diseases and the adaptation and evolution of natural populations^44^. Since NOIA overcomes the duality of functional and statistical models of epistasis, it serves as a previously validated means to obtain estimates of both functional and statistical genetic effects from data. Moreover, explicit orthogonality is achieved regardless of the genotype frequency, making it an optimal tool for assigning polygenic loci to complex traits in relatively small populations^44^. We performed NOIA analysis as a three loci model including genotypes of the two *APOB* and one *PLA2G4A* SNPs in association with each of the 37 phenotype measurements. NOIA identified 62 significant (*p* < 0.05) models across 15 phenotypes (Supplemental Table S8). Of the significant models, 23 showed two loci having an effect on the phenotype suggesting a significantly robust polygenic effect. Genotype-phenotype plots were generated from the most significant model for each phenotype. Selected plots are shown in Fig. 3 and the remainder of the plots are in Supplemental Fig. 3.

Figure 3 projects six of the 37 selected genotype-phenotype NOIA associations in three loci models based on genotypes from the extended panel of high fat diet exposed Japanese macaques (*n* = 43 animals). The *Genotype* (x-axis, with 1 annotating the homozygous major allele, 2 the heterozygous state, and 3 the homozygous minor allele) shows the genotypes for each of the loci (ordered as *PLA2G4A*, *APOB*.1, and *APOB*.2). Significance of association is annotated by boxing, with the genotypes and allelic variation predicted to associate with the phenotype in a significant manner being outlined with colored boxes. The *Genotypic Effects* (y-axis) shows the NOIA predicted effect of the genotype on a phenotype, with the value being specific to the phenotype. For example, in panel Fig. 3a, weight change as a percentage of baseline (ranging from −50% to 100%) is projected on the y axis. In this model, locus 2 with *APOB.1* is demonstrated to be significant in an additive model with a *p* = 0.035. In contrast, neither *APOB.2* nor *PLA2G4A* demonstrated a significant effect on weight change (one phenotypic characteristic which renders of fails to render obesity). In fact, when examining all six other phenotypic characteristics projected in Fig. 3, the *APOB.*1 locus demonstrated an additive effect on weight change percent (Fig. 3a), leptin/body weight ratio (Fig. 3b), and total fat based on DXA (Fig. 3c). By comparison, the *PLA2G4A* (Fig. 3d) and *APOB*.2 loci show a combined additive effect on insulin/glucose ratio. Similarly, the *PLA2G4A* locus shows an additive effect on triglyceride levels (Fig. 3e) while the *APOB*.2 locus shows an additive effect on free fatty acid levels (Fig. 3f). This is summarily and systematically repeated with 9 of the 37 phenotypic measures demonstrating significance by NOIA projected in Supplemental Figure S3.

## Discussion

We applied comparative principles and designed an exon capture array of conserved metabolism genes for use in Japanese macaque, and demonstrated the capacity of this technology to identify functional SNPs related to insulin sensitivity and resistance to obesity in a relevant primate model of chronic high fat diet exposure. We discovered that although body weight and body composition traits are quantitative, two loci in *APOB* and one in *PLA2G4A* withstood complex modeling across multiple traits. By application of explicit orthogonal modeling (NOIA), we estimated the polygenic and interactive nature of these loci against multiple traits which collectively render an obesity resistant phenotype in our adult female dams.

Our approach towards identifying obesity resistant loci in adult primates has significant advantages and provides clarity over previous human GWAS analysis seeking to identify susceptibility loci. Recent meta-analyses^2^,^3^ identifying specific loci and common variants accounting for up to 21% of BMI variation only nominally predict obesity or waist-to-hip ratio (WHR) in women with relatively modest effect size estimates (per allele OR 1.2–1.6)^2^,^12^,^15^. One possible reason for such low predictive capacity in human GWAS analyses arises from the inherent heterogeneity of potentiating factors in human studies. Large GWAS studies do not retain detailed phenotypic nor trait data on individual subjects, and cannot control for variation in dietary caloric intake nor high fat density, do not control for exercise, nor reliably estimate temporal variation based on life stage.

We have overcome these inherent limitations to human GWAS analysis in our well-characterized primate model in three manners. First, we challenged all animals in our analysis to a high fat diet and normalized our trait measures to reproductive life-stage, time of exposure to the diet, and baseline characteristics at the time of initial diet allocation. Second, we estimated association with 37 different trait loci which have both independent and dependent predictive capacity and estimated their interaction. This second manner enabled us to further identify significant associations with multiple additional traits that accompany or lend to the obesity resistant phenotype in an extended panel of all 43 adult female dams on the HFD for at least 3 years with extensive phenotype data. Third, we were thus able to apply multiple previously validated means for fine-mapping of polygenic and interactive loci.

From a translational perspective, the *APOB* C/T SNP at chr13:20932165 coding for glycine or aspartic acid is of particular interest because it is also present in humans as SNP rs142828185. Although little information is presently available regarding the SNP with no minor allele frequency or phenotypic association reported in dbSNP, much is known about the role of APOB as a primary apolipoprotein of LDLs^45^,^46^. Specifically, familial hypobetalipoproteinemia is a co-dominant disorder characterized by low plasma levels of LDL cholesterol due to coding and splice site mutations in *APOB*^47^. The splice site mutations result in truncated APOB protein, which causes plasma secretion defects leading to fatty liver disease^47^. However, such mutations are rare, making our finding of a C/T SNP with a modeled impact on multiple measured traits of particular interest.

Because we found that the *APOB* C/T SNP at chr13:20932165 coding for glycine or aspartic acid was associated with multiple traits in addition to levels of serum LDL, the likelihood that these and potentially other variants in humans would culminate in up to one third of adult females being resistant to high fat diet induced obesity is both novel and of particular interest. Such pleiotropy would support a model of gene-by-food environment interactions, whereby the majority of a population cohort are susceptible to HFD with resultant insulin resistance and obesity. To this end, and of particular interest to our study, common coding variants in *APOB* have recently been suggested to play a role in maternally-derived obesity in young adults. Specifically, Hochner *et al*. recently demonstrated that a common coding variant in the 4th exon of *APOB* (rs1367117) demonstrated a significant maternally-derived effect on BMI (β 0.8; 95% CI:0.4,1.1; *p* 3.1 × 10^−5^) and waste circumference (β 2.7; 95% CI:1.7,3.7; *p* 2.1 × 10^−7^)^48^. By contrast, corresponding paternally-derived effects were non-significant (*p* > 0.6). Moreover, these investigators working with 1250 young adults and their parents were able to demonstrate a dominant maternal parent-of-origins effect, suggestive of maternally-derived associations of rs1367117 with elevated fasting glucose (β = 0.9; 95% CI:0.3,1.5; *p* 4.0 × 10^−3^) and insulin (ln-transformed, β = 0.06; 95% CI: 0.03,0.1; *p* 7.4 × 10^−4^)^48^. In a second recent study of Korean adults, the investigators analyzed the effects of *APOB* rs1469513 on obesity-rendering traits, alongside the interaction of *APOB* rs1469513 and dietary intake on these same traits^49^. They found that plasma levels of total cholesterol (*p* 0.001) and low-density lipoprotein cholesterol (*p* 0.010), body weight (*p* 0.048), and body mass index (*p* 0.029) were significantly different in carriers of the A allele and minor G allele of *APOB* rs1469513. Among individuals whose fat intake was above the median of the study cohort, the difference for the body mass index across genotypes was 1.14% (AA allele 24.66 kg/m^2^ versus AG+GG 24.94 kg/m^2^, *p* 0.004) and carriers of the minor G allele had increased odds of being obese (odds ratios, 1.31; 95% CI 1.09, 1.57; *p* 0.004) compared with homozygotes for the A allele^49^. What we have observed in the current study is a similar significant impact of another variant of the *APOB* gene demonstrating an alternate risk of modifying risk of obesity resistance. What is notable is the concomitant impact of both presumptive maternal parent of origin effect, as well as modifier of dietary impact, in our primate cohort. Taken together, our findings alongside the current collective literature suggests that *APOB* is a primary determinant of high fat diet induced obesity, and may render multigenerational impact through a maternal parent of origin effect.

*PLA2G4A* is of additional translational interest, although less is known regarding variants in the human population. The *PLA2G4A* gene encodes a key calcium activated enzyme which catalyzes the hydrolysis of membrane phospholipids to release arachidonic acid, rendering eicosanoids (prostaglandins and leukotrienes) and lysophospholipids^47^. In a recent clinical trial investigating the role of 6 weeks of fish oil supplementation and SNPs in *PLA2G4A* on plasma triglyceride levels in 208 human subjects, genotype by supplementation interaction effects were observed for 4 SNPs. Specifically, the rs10752979, rs10737277, rs7540602, and rs3820185 in *PLA2G4A* withstood MIXED procedure testing adjusting for age, sex, BMI and energy intake. NOIA generated four models showing an association between *PLA2G4A* genotype and triglyceride levels including two models in which *PLA2G4A* was dominant (Fig. 3a and Supplemental Table S8). Our findings in the non-human primate support emerging data from human studies that polygenic variants in lipid metabolism (*APOB*) and central enzymes for mediating eicosanoid-driven inflammation (*PLA2G4A*) modulate obesity-resistant traits in response to dietary challenge or supplementation^47^.

Equally intriguing are these current findings in context with our prior demonstrations that generally regardless of maternal insulin resistance and obesity status, there is a notable impact of high fat diet feeding during gestation and lactation among offspring. The impact of early in life high fat diet exposure are noteworthy and include non-alcoholic fatty liver disease (NAFLD) with lipid accumulation, pancreatic and immune modulatory dysfunction, thyroid hormone dysregulation, hypertension, and alterations in sirtuins and their regulated pathways^18^,^27^,^50^,^51^,^52^. These further manifest as aberrant metabolic homeostasis and altered behaviors at both 1 and 3 years of age^30^,^31^, with early increased body mass trajectories. Many of these metabolic characteristics persist despite switching to a healthy diet after weaning. When our prior observations are put into context with our findings reported here, these data collectively suggest that in a first generation exposed to a HFD, genomic loci differentially regulate trait propensity measured as obesity and insulin resistance. However, in a second generation (their offspring), the weighted effect of the high fat diet exposure during gestation and lactation may be dominant. As discussed above, it is unclear whether this is the result of variation in genomic susceptibility, rendered maternal parent of origin effect^48^, or alternately a dominance of epigenomic modifications in key gene regulatory events. Given our prior observations regarding offspring epigenomic reprogramming and persistent intestinal dysbiosis (gut microbiome variation), it is intriguing to speculate on the potential relative effect size measures of such modifiable events^19^,^24^,^25^,^26^. Based on these collective phenotypic and molecular observations in primates, we speculate that while there may be genomic-mediated variation in obese resistance to chronic high in a first generation (the dams), the same would not hold true for their offspring as a result of maternal diet-induced epigenomic and metagenomics modifications in the offspring. Were this to similarly hold true in human populations, we would anticipate observing an increased rate, prevalence, and point incidence of obesity with earlier onset (anticipation) in each generation exposed to a chronic high fat maternal diet. In fact, this has been well described and is supportive by the preponderance of epidemiologic data in human population based cohorts^1^. However, in view of the results of others suggesting that a distinct loci in *APOB* in humans may render susceptibility to obesity in first generation young adult offspring^48^, further studies are needed to firmly establish relative genomic effect size estimates.

The loci identified here as significant represent an experimentally robust, in-depth, fine-mapped genotype-phenotype association analysis in a highly relevant and well-characterized primate model. Future application of our findings into human cohorts are likely to yield important insights into the molecular and physiologic mechanisms leading to obesity and insulin resistance in not only the current generation, but in the generations to come.

## Methods

### Exon hybrid capture array design, application for variant calls, and genotype validation

A total of 783 lipid metabolism, obesity, and fatty acid related genes were selected as targets (Supplemental Table S9). Human, chimpanzee, orangutan and rhesus macaque genome assemblies were used to identify 85 bp sliding windows within coding exons with 90% minimum conservation (Supplemental Figure 1). Genome-wide frequencies of 15mers in rhesus windows were calculated, and windows with an average 15 mer frequency <100 were used to design oligo probes for the array (NimbleGen). Genomic DNA was extracted from Japanese macaque dams (n = 8) identified after 3 years as being high fat diet resistant or sensitive by mean % bodyweight gain (22.3 vs 66.5%), leptin/body weight ratio (0.08 vs 0.79), and insulin AUC (2589 vs 10210 mU/ml). Fragments were hybridized to the array and bound fragments were released and sequenced (454 FLX Titanium). Functional SNPs of interest were identified (Supplemental Figure 2) and sequenced in an external validation cohort comprised of a total of 71 study dams (resistant, sensitive or control) with deep phenotyping data.

### Non-human primate model of maternal chronic high fat diet feeding

All animal protocol work is approved by and in accordance with the relevant guidelines and regulations as stated by the institutional animal care and use committees (IACUC) at both Baylor College of Medicine (protocol number AN-4752) and the Oregon National Primate Research Center (ONPRC). *Experimental design:* The use of *M. fuscata* by our consortium of investigators has been previously described^24^,^25^,^26^,^27^,^28^,^29^,^30^,^31^,^32^,^33^. In brief, animals are socially housed (4–9 females and 1–2 males per group) within indoor/outdoor enclosures at the Oregon National Primate Research Center (ONPRC). Animals were on an isocaloric diet and were either fed a control diet of standard chow (Monkey Diet no. 5000; Purina Mills Co., St. Louis, MO) consisting of 14.6% calories from fat (soya bean oil) or high-fat diet (TAD Primate Diet 5LOP, Test Diet, Richmond, IN) consisting of 36.6% calories from fat (lard, butter, animal fat, and safflower oil) that is additionally supplemented with caloric dense treats. Animals are initiated on their allocated diet prior to mating, and then maintained on their allocated diet through duration on study (up to 9 years), except when otherwise indicated. Body weight and baseline insulin, glucose, and triglyceride measurements were collected, and intravenous glucose tolerance tests (GTTs) were performed uniformly during the 3^rd^ trimester of pregnancy or post lactation/pre-gestation. The area under the curve (AUC) for glucose and insulin were calculated from zero values. Body composition analysis (DXA) was performed in year 3 of diet exposure in the non-pregnant state, and lean and fat mass were calculated with standard measures has been previously described^24^,^25^,^26^,^27^,^28^,^29^,^30^,^31^,^32^,^33^. Plasma glucose, insulin, non-esterified fatty acids, triglycerides, HOMA-IR and all other laboratory values were measured or calculated as previously described^24^,^25^,^26^,^27^,^28^,^29^,^30^,^31^,^32^,^33^. Animals bred naturally, and offspring (both male and female) were maintained on a similar diet as their mothers until weaning (6–8 months) when the offspring either became part of the control cohort (maintaining the same diet as their mothers’) or the crossover cohort (switching the diet from their mothers). Offspring were not employed in the study described herein, and descriptions are included only to emphasize ongoing lactation and maintenance of offspring-maternal pairing through weaning.

### Phenotyping

The full extended trait panel is shown in Table S4, and includes quantitative lipid profiles (total cholesterol, LDL, HDL, trigylcerides, free fatty acids), glucose, insulin and HOMA-IR, weight, BMI, and DXA values with calculated weight change/years on diet. Additional testing and derived values include GTT, leptin, glucagon, and thyroid hormone testing. These values, their ratios, and derived calculations (inclusive of AUC and HOMA-IR) were thereafter employed in both raw and matrix testing as described; significant associations are shown in Figures and Supplemental Figure 3 and Table S5. All testing procedures were performed in a uniform fashion on fasting dams and as previously described^20^,^21^,^22^,^23^,^24^,^25^,^26^,^27^,^28^,^29^,^30^,^31^,^32^,^33^.

### Statistical analysis

Our initial SNP calls were based on infinite odds ratios (*i.e.,* present in all 4 dams of one group, and absent in all of the other) as estimated by ssahaSNP, which only identifies homozygous alternative allele SNPs. In order to further assure high quality and statistically robust SNP identification in an initial small discovery cohort of 8 animals, three tools were used to call SNPs (Atlas-SNP2^35^, SNPTools^36^, and ssahaSNP^37^) and the only SNPs called by all three tools were selected as candidates for further analysis. Variant Effect Predictor (VEP)^38^ based on rhesus gene models was used to identify functional consequences of the SNPS and SNPs were lifted over to the human genome in order to perform SIFT^39^ and PROVEAN^40^ predictions of the effect of amino acid differences. PLINK^41^ was used to identify associations between the genotypes with phenotypes. Nonsynonymous SNPs with SIFT or PROVEAN predicted effects on protein function, and PLINK adaptive permutation empirical *p* values < 0.063 were identified for further analysis. Combined Annotation Dependent Depletion (CADD)^42^ was employed to integrate multiple annotations and predict the likelihood of a SNP having a functional consequence. A PHRED score of > = 20 indicates the SNP is among the 1% of SNPs in the human genome most likely to have a functional effect; a PHRED score > = 10 indicates prediction to be among the 10% most likely SNPS to have a functional effect. Application of the Natural and Orthogonal InterAction (NOIA)^44^ model to the extended panel was used to refine genotype-phenotype associations suggested by PLINK analysis of SNPs identified by the exon capture arrays. NOIA analysis was performed as a three loci model including genotypes of the two *APOB* and one *PLA2G4A* SNPs in association with each of the 37 phenotype measurements with significance defined by probability (*p* < 0.05) models across 15 phenotypes.

## Additional Information

**How to cite this article**: Harris, R. A. *et al*. Genomic Variants Associated with Resistance to High Fat Diet Induced Obesity in a Primate Model. *Sci. Rep.* **6**, 36123; doi: 10.1038/srep36123 (2016).

**Publisher’s note:** Springer Nature remains neutral with regard to jurisdictional claims in published maps and institutional affiliations.

## Supplementary Material

Supplementary Information

Supplementary Tables
