# Supplementary material for: Genomic Variants Associated with Resistance to High Fat Diet Induced Obesity in a Primate Model
Source: Sci Rep. 2016 Nov 4;6:36123. doi: 10.1038/srep36123 (PMC5095882; doi:10.1038/srep36123)
Supplement: Supplementary Information [file srep36123-s1.pdf]

Supplemental Information for:

Genomic Variants Associated with Resistance to High Fat Diet Induced Obesity in a Primate Model

R. Alan Harris PhD[1,2], Callison E. Alcott[3], Elinor L. Sullivan PhD[4,5], Diana Takahashi[4], Carrie E. McCurdy PhD[6], Sarah Comstock PhD[7], Karalee Baquero[4], Peter Blundell[4], Antonio E. Frias, MD[4,8], Maike Kahr MD[1], Melissa Suter PhD[1], Stephanie Wesolowski PhD[9], Jacob E. Friedman PhD[9], Kevin L. Grove PhD[4], and Kjersti M. Aagaard MD PhD[§1,2,3,4,10]

Affiliations:
[1]Department of Obstetrics & Gynecology, Division of Maternal-Fetal Medicine at Baylor College of Medicine and Texas Children's Hospital, Houston, TX.
[2]Department of Molecular and Human Genetics at Baylor College of Medicine, Houston, TX.
[3] Developmental Biology Interdisciplinary Program at Baylor College of Medicine, Houston, TX.
[4]Oregon National Primate Research Center, Oregon Health & Science University (OHSU), Beaverton, OR.
[5] Department of Biology, University of Portland
[6]Department of Human Physiology, University of Oregon, Eugene, OR.
[7]Department of Biology, Corban University, Salem, OR.
[8]Department of Obstetrics & Gynecology, Division of Maternal-Fetal Medicine, OHSU, Portland, OR.
[9]Departments of Pediatrics, University of Colorado Anschutz Medical Campus, Aurora, CO.
[10]Department of Molecular and Cell Biology at Baylor College of Medicine, Houston, TX.

[§]Correspondence to:
Kjersti Aagaard, M.D. Ph.D.
Baylor College of Medicine
Department of Obstetrics & Gynecology
Houston, TX 77030
aagaardt@bcm.edu

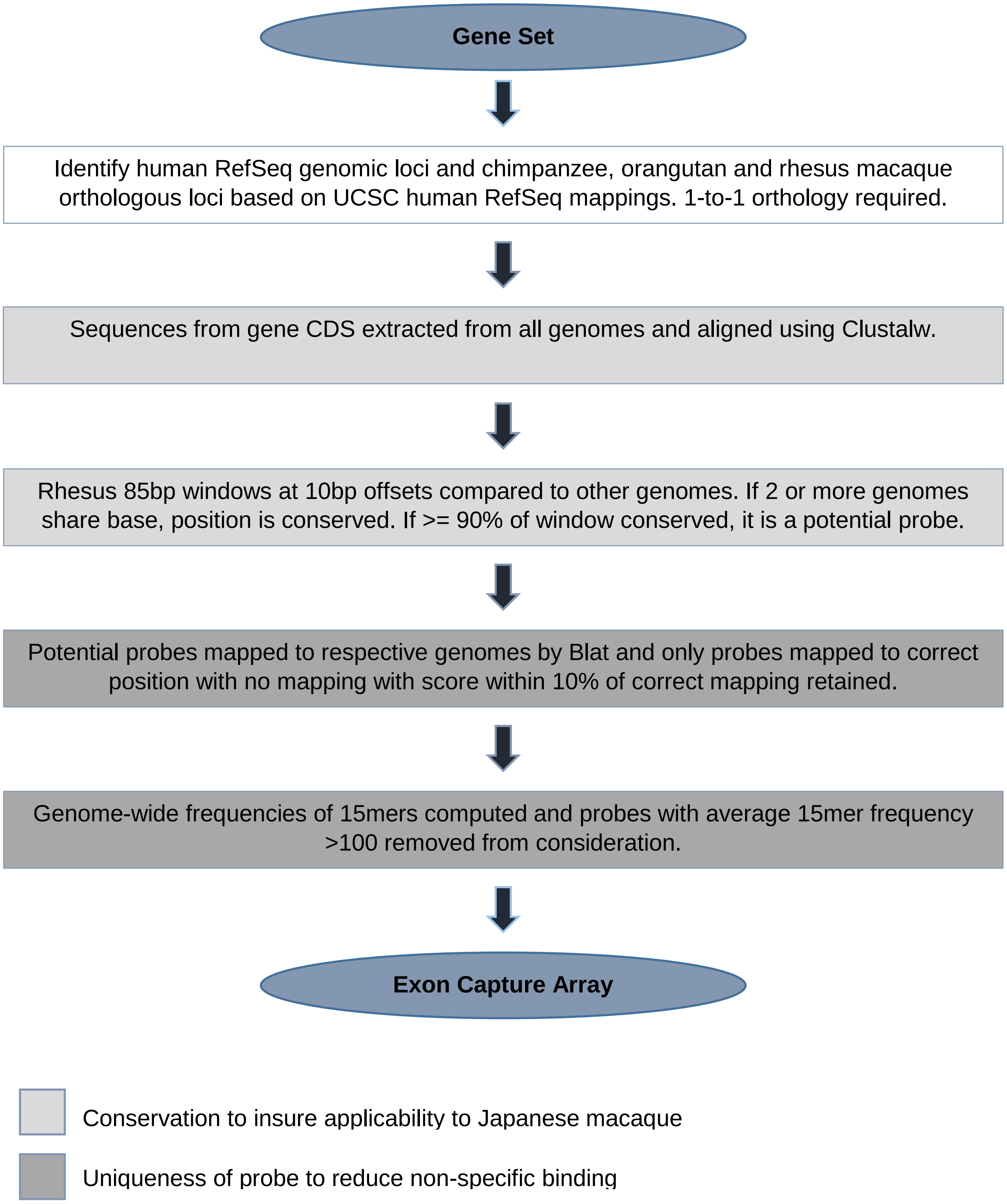

Supplemental Figure 1. Exon capture array design.

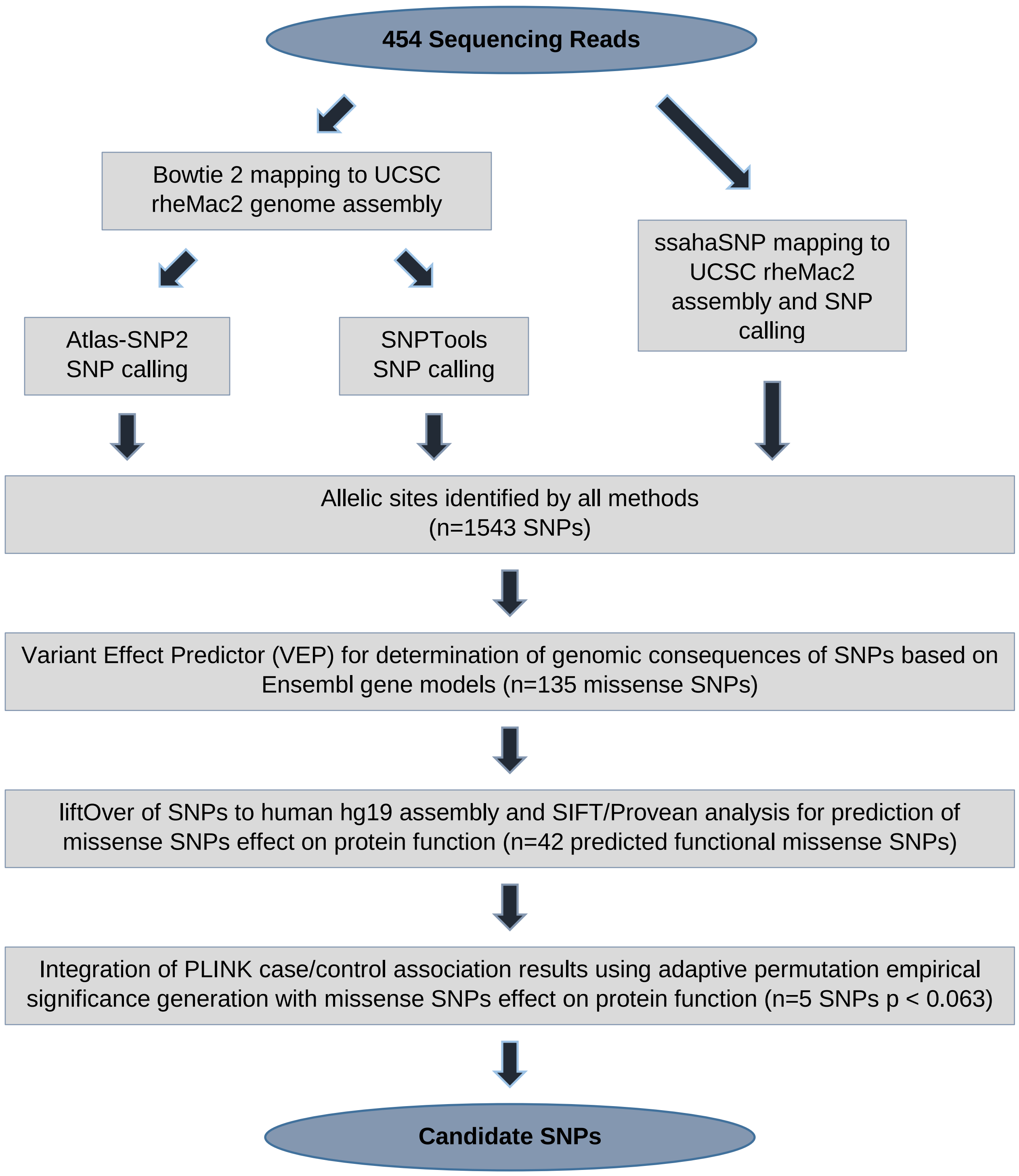

Supplemental Figure 2. SNP Calling

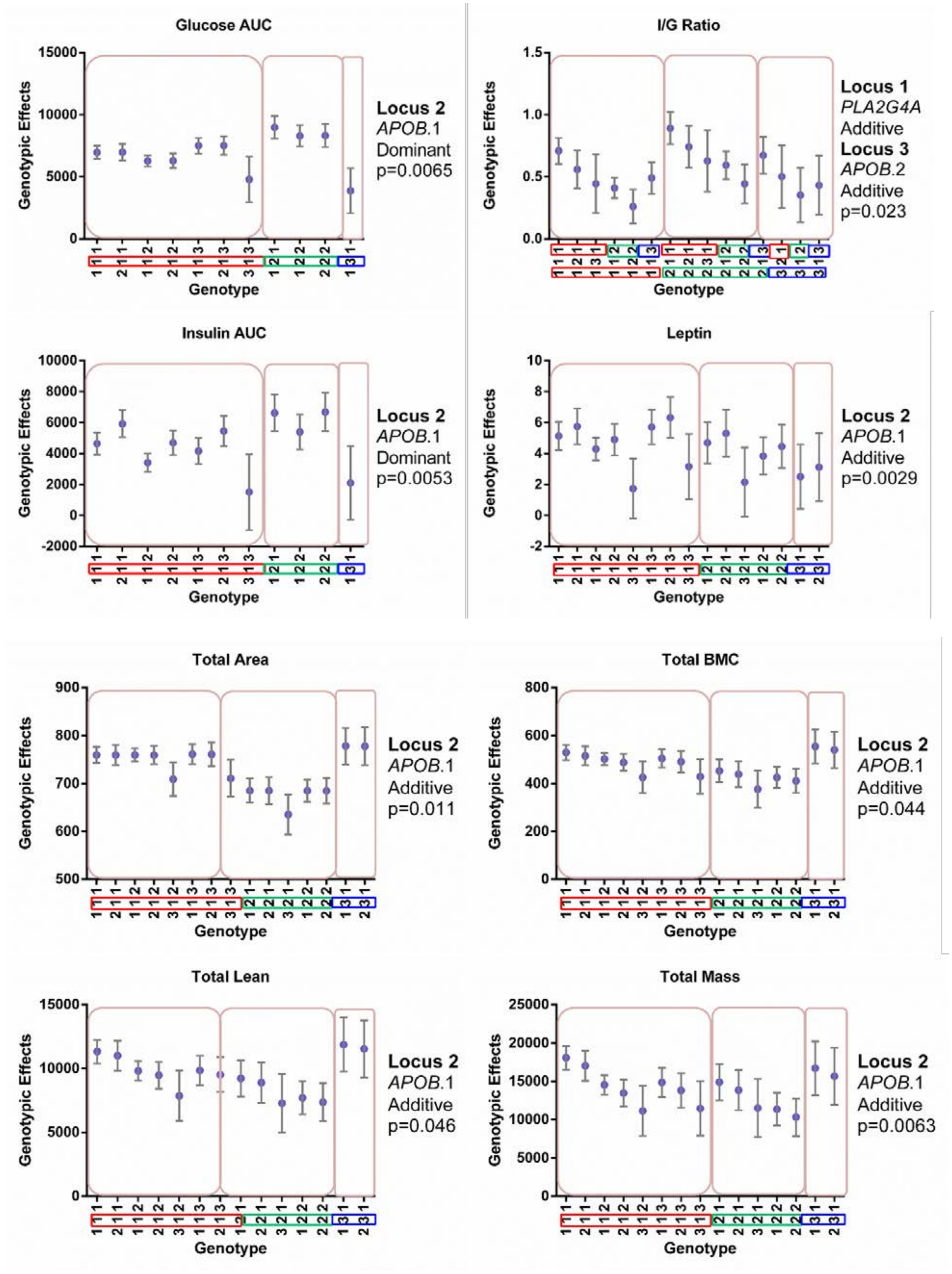

Glucose AUC
Genotypic Effects
Genotype
Locus 2
APOB.1
Dominant
p=0.0065
I/G Ratio
Genotypic Effects
Genotype
Locus 1
PLA2G4A
Additive
Locus 3
APOB.2
Additive
p=0.023
Insulin AUC
Genotypic Effects
Genotype
Locus 2
APOB.1
Dominant
p=0.0053
Leptin
Genotypic Effects
Genotype
Locus 2
APOB.1
Additive
p=0.0029
Total Area
Genotypic Effects
Genotype
Locus 2
APOB.1
Additive
p=0.011
Total BMC
Genotypic Effects
Genotype
Locus 2
APOB.1
Additive
p=0.044
Total Lean
Genotypic Effects
Genotype
Locus 2
APOB.1
Additive
p=0.046
Total Mass
Genotypic Effects
Genotype
Locus 2
APOB.1
Additive
p=0.0063

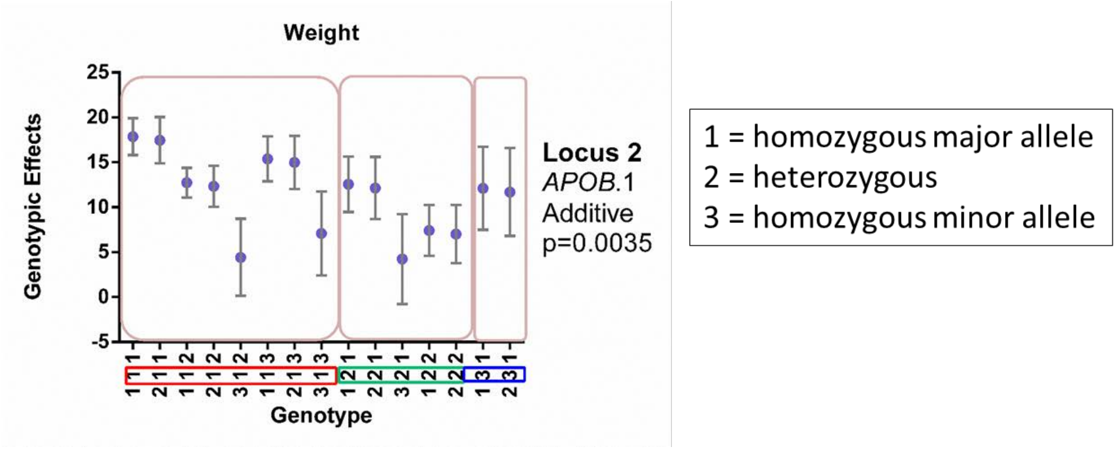

Supplemental Figure 3. Significant ($p < 0.05$) genotype-phenotype NOIA three loci models based on genotypes for the extended panel of high fat diet exposed Japanese macaques. The Genotype axis shows the genotypes (1 = homozygous most frequent allele, 2 = heterozygous, 3 = homozygous least frequent allele) for each of the loci (ordered as *PLA2G4A*, *APOB*.1 *APOB*.2) and the Genotypic Effects axis shows the NOIA predicted effect of the genotype on a phenotype.
